# Periosteal Pedicle Flap Harvested during Vestibular Extension for Root Coverage

**DOI:** 10.1155/2015/124039

**Published:** 2015-12-16

**Authors:** Shubham Kumar, Krishna Kumar Gupta, Rahul Agrawal, Pratima Srivastava, Shalabh Soni

**Affiliations:** ^1^Department of Periodontics, Sardar Patel Post Graduate Institute of Dental & Medical Sciences, Lucknow, Uttar Pradesh 226025, India; ^2^Department of Periodontics, Vyas Dental College & Hospital, Jodhpur, Rajasthan 342003, India

## Abstract

Root exposure along with inadequate vestibular depth is a common clinical finding. Treatment option includes many techniques to treat such defects for obtaining predictable root coverage. Normally, the vestibular depth is increased first followed by a second surgery for root coverage. The present case report describes a single-stage technique for vestibular extension and root coverage in a single tooth by using the* Periosteal Pedicle Flap (PPF)*. This technique involves no donor site morbidity and allows for reflection of sufficient amount of periosteal flap tissue with its own blood supply at the surgical site, thus increasing the chances of success of root coverage with simultaneous increase in vestibular depth.

## 1. Introduction

Gingival recession is defined as an apical shift of the gingival margin with exposure of root surface to the oral cavity [1]. Gingival recession displaces the gingival margin apically with shallowing of the width of attached gingiva and reducing of the vestibular depth. A narrow zone of attached gingiva is believed to be insufficient to protect the periodontium from any type of injury resulting from friction forces generated during mastication and pull forces created by the muscles of the adjacent alveolar mucosa on the gingival margin [2].

Successful coverage of exposed roots for esthetic and functional reasons has been the objective of various mucogingival procedures. Many techniques have successfully been utilized for root coverage; however a graft that has its own blood supply, which can be harvested adjacent to the defect in sufficient amounts without requiring any second surgical site and has the potential for promoting the regeneration of lost periodontal tissue, is a long-felt need [3].

The periosteum is a highly vascular connective tissue sheath covering the external surface of all the bones except sites of articulation and muscle attachment [4]. The adult human periosteum is known to contain fibroblasts and their progenitor cells which retain the ability to differentiate into fibroblasts, osteoblasts, chondrocytes, adipocytes, and skeletal myocytes. The tissues produced by these cells include cementum with periodontal ligament fibers and bone [5]. The availability of sufficient periosteum adjacent to recession defect and its utilization as a graft for root coverage was first described by Gaggl et al. (2005) [6].

The present case report describes a technique where vestibular extension by fenestration technique was performed and the layer of periosteum reflected after fenestration was used as a pedicle flap for root coverage.

## 2. Case Report

**Figure 1:**
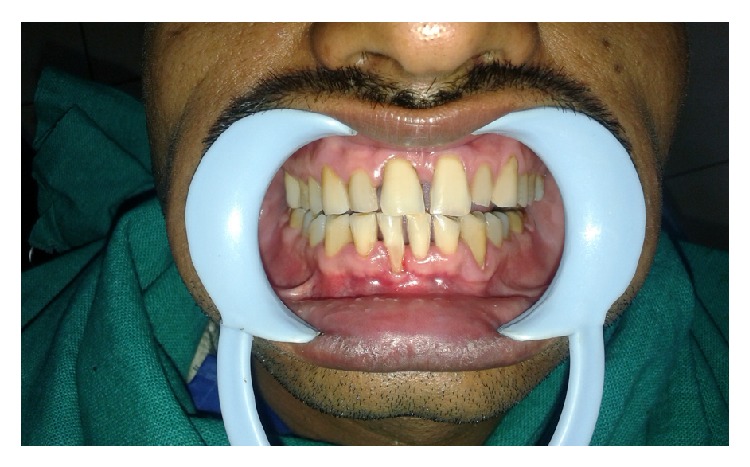
Preoperative view.

**Figure 2:**
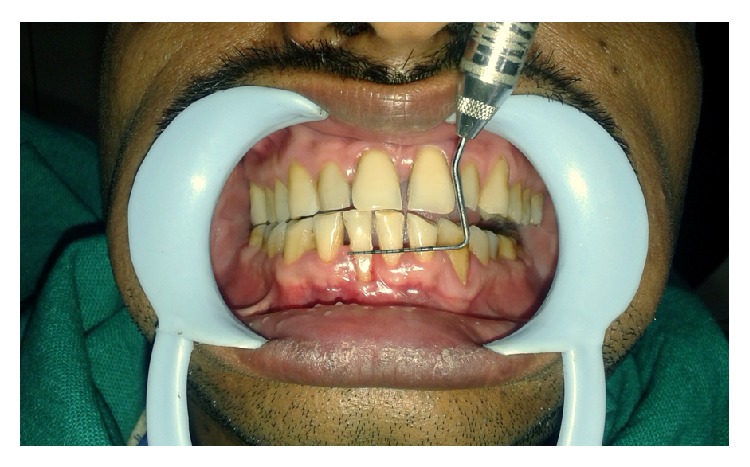
Miller's Class II recession with 4 mm wide defect.

**Figure 3:**
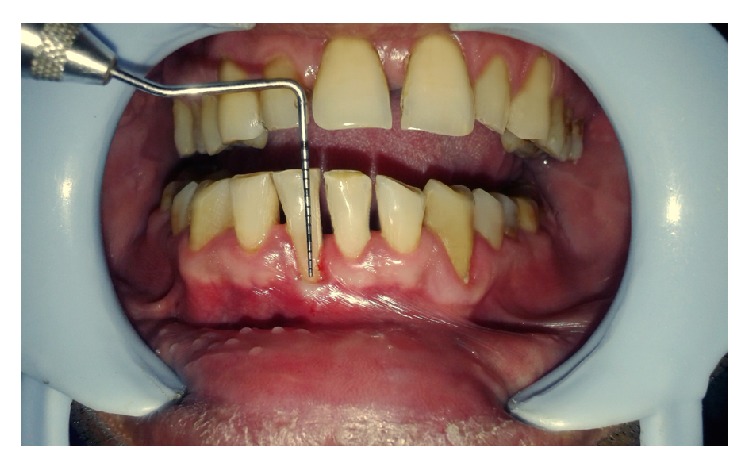
Miller's Class II recession with 5 mm deep defect.

A 32-year-old male patient reported to the Department of Periodontics with the chief complaint of receding gums in lower front tooth region (Figure 1). On examination, a 4 mm wide and 5 mm deep Miller Class II gingival recession was found in right mandibular central incisor (Figures 2 and 3). The tooth was nonmobile and patient also gave the history of tooth brush trauma. The vestibular depth and the width of attached gingiva were also inadequate in the region. Thorough general assessment of the patient was made by case history recording, clinical examination, and routine laboratory blood investigations. The patient was in good systemic health with no contraindications for periodontal surgery. The patient received phase I therapy and the surgery was planned after three weeks. The patient was also given a hard acrylic maxillary night guard appliance for overnight use to prevent the delirious effect of bruxism on the masticatory system.

**Figure 4:**
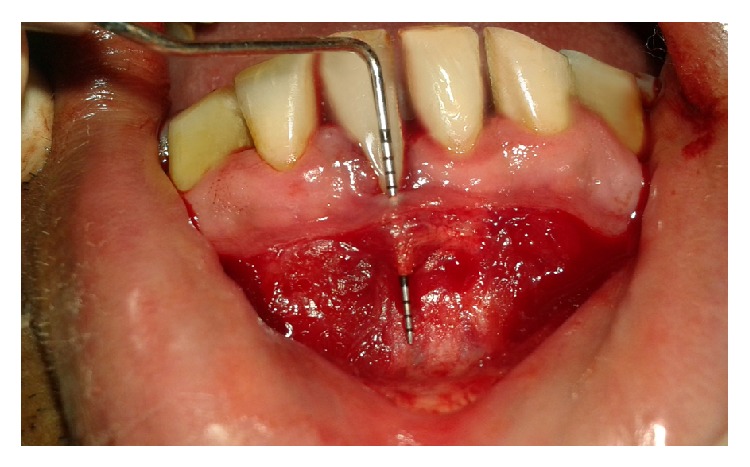
Tunnel preparation.

After extraoral mouth preparation (Betadine 10%) and intraoral mouth preparation (10 mL of 0.2% Chlorhexidine for 1 minute), bilateral mental nerve block (Lignocaine 2% with 1 : 80,000 Adrenaline) was given. A horizontal incision was made using a Number 15 Bart Parker (BP) blade at the mucogingival junction from left mandibular canine to right mandibular canine retaining all of the attached gingiva. A partial thickness flap was reflected by sharply dissecting muscle fibres and tissue from the underlying periosteum. The gingival recession defect site/recipient site was prepared by apical extension of the crevicular incision along the right mandibular central incisor with split thickness dissection of the facially located tissues up to the level of the vestibular incision so as to create a tunnel (Figure 4) using Number 15 BP blade. A reverse bevel incision was made all along the soft tissue margin of the recession defect.

**Figure 5:**
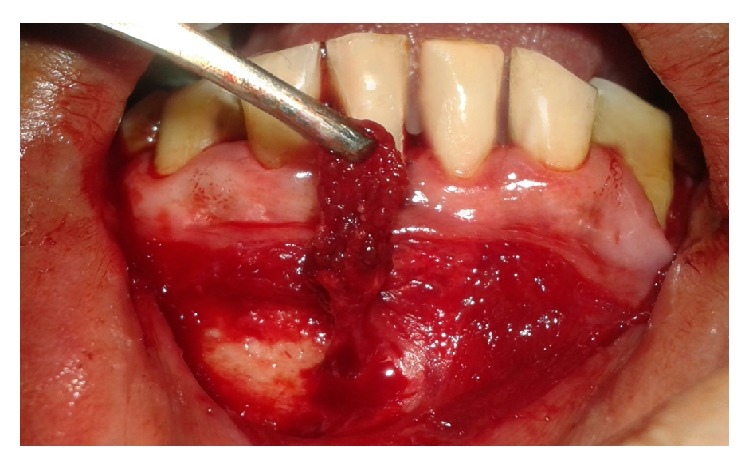
Periosteal Pedicle Flap raised by incomplete fenestration.

**Figure 6:**
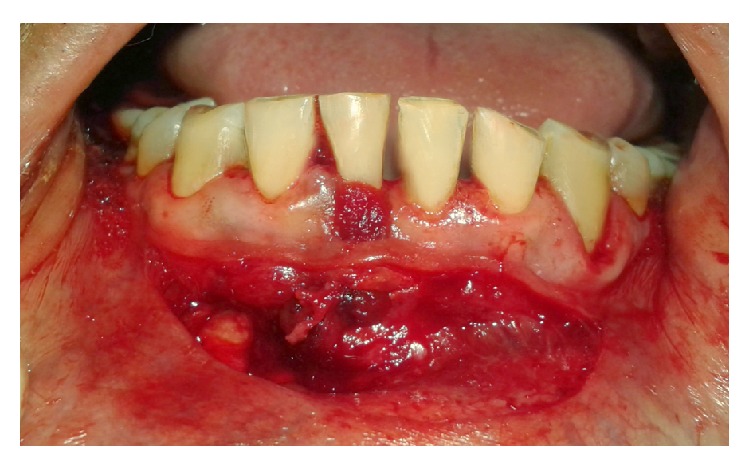
Periosteal Pedicle Flap placed on the area of recession via tunnel.

**Figure 7:**
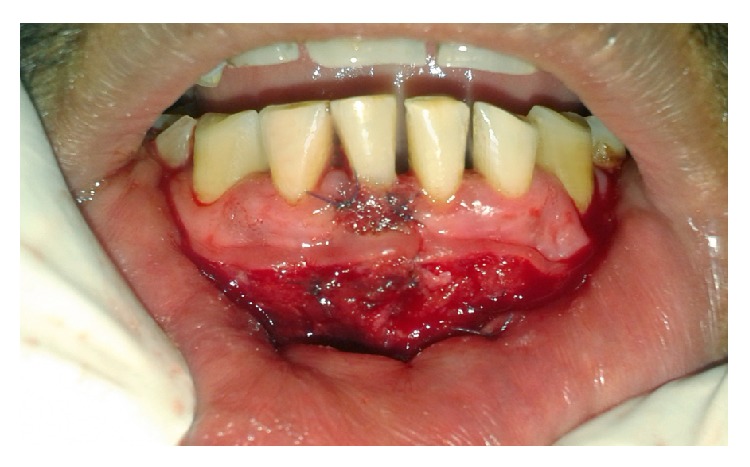
Suturing done using 5-0 resorbable sutures.

A strip of periosteum was then removed at the level of the mucogingival junction, causing a periosteal fenestration and exposing the underlying bone. Care was taken not to remove the periosteal strip completely and to leave it attached to the bone and the rest of the surrounding periosteum at the contralateral end (Figure 5). Thus, the periosteum remained pedicled at one end and hence the name* “Periosteal Pedicle Flap”* is given. The exposed root surface was planned with Columbia #2R-2L universal curette (Hu-Friedy) and was biomodified using Tetracycline HCl in a ratio of 100 mg/mL for 3 minutes. The area was thoroughly irrigated and the PPF was then repositioned vertically towards the recession area, passing through the tunnel (Figure 6). While the PPF was sutured to the recipient bed, the labial mucosa was sutured apically to the periosteum at the depth of the fenestration using resorbable 5-0 sutures (Vicryl, Ethicon) (Figure 7).

**Figure 8:**
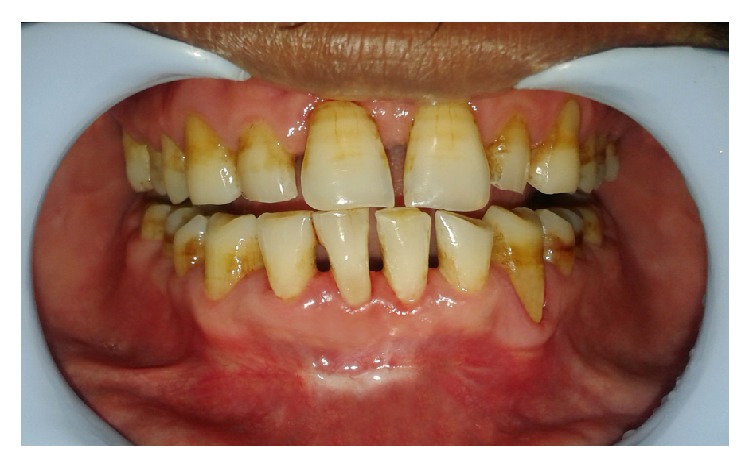
6-month postoperative view.

Periodontal dressing (Coe-Pak; GC America Inc.) was applied over the surgical area and medication was prescribed for 5 days that included Amoxicillin 500 mg, TDS, Paracetamol 500 mg + Aceclofenac 100 mg, BD, and Probiotics, OD. Tooth brushing was discontinued for the first 2 weeks at the surgical site and 10 mL 0.2% chlorhexidine mouth rinse twice daily was instructed till 4 weeks after surgery. Coe-Pak was removed 10 days after the surgery and the patient was asked to maintain meticulous oral hygiene. Healing was uneventful and was nearly complete, with minimal postoperative discomfort by 3rd week. The recipient site showed adequate coverage with minimal probing depths and a favourable esthetic result after 6 months (Figure 8).

## 3. Discussion

The indications for surgical treatment of gingival recession include reducing root sensitivity, minimizing cervical root caries, increasing the zone of attached gingiva, and improving esthetics. Miller (1987) defined complete root coverage as the location of soft tissue margin at the cementoenamel junction, presence of clinical attachment to the root, sulcus depth of 2 mm or less, and absence of bleeding on probing [7]. However, Wennström and Zucchelli (1996) suggested that an increase in gingival height independent of the number of millimetre is considered as a successful outcome of gingival augmentation procedures [8]. Despite numerous techniques available for the treatment of gingival recession defects, no single universal technique can be used with high predictability, effectiveness, and efficiency.

The periosteum is a highly vascular tissue and is comprised of 2 layers: the inner cellular or cambium layer that contains numerous osteoblasts and osteoprogenitor cells [9] and an outer fibrous layer composed of dense collagen fibre and fibroblasts and their progenitor cells [10]. The periosteum has a rich vascular plexus, and a study showed that periosteal cells release vascular endothelial growth factor and induces angiogenesis [11]. Periosteum, the* “Sleeping Giant”* springs into action by surgical trauma and provides “*a river of regenerative tissue*”* moving* centripetally into the wound thus favouring fibrogenesis and osteogenesis. It also acts as a springboard for nerve regeneration into overlying gingiva, mucosa, or graft [12].

Wound healing after mucogingival surgery relies on clotting, revascularization, and maintenance of blood supply. Also, a vascular graft is more likely to survive on an avascular root surface. These qualities make the periosteum a suitable graft over an avascular root surface. In addition, having an adequate vascularity prevents its necrosis even if it is left uncovered by the overlying flap, especially in the case of a large area of gingival recession.

Rajpal et al. [13] and Shah et al. [14] reported similar cases where the Periosteal Pedicle Flap reflected during vestibular extension was used as a pedicle flap for root coverage in single tooth with Miller's Class II recession. The result obtained in our case was similar to the result obtained in these cases. Mahajan reported successful treatment of multiple gingival recession defects utilizing Periosteal Pedicle Graft [3].

The regenerative potential of the periosteum has been demonstrated by many studies. Lekovic et al. [15] and Verma et al. [16] successfully used periosteum as a barrier membrane for treating Grade II furcation defects. Kumar et al. reported better regeneration with alloplastic graft material utilizing periosteum as barrier membrane [17]. Singhal et al. used Periosteal Pedicle Graft in two-wall intrabony defects and reported 48.88% decrease in bone defect area after 6 months [18].

The procedures employing Periosteal Pedicle Flap are usually well tolerated by patient with minimal postoperative discomfort; however cases with postoperative extraoral swelling and ecchymosis have also been documented [19]. Such complications can be limited by gentle tissue handling, minimizing the duration of surgery, avoiding overt flap reflection, and ensuring adequate haemostasis before suturing.

The advantages of this technique over other techniques are

- vestibular deepening and root coverage achieved in a single procedure;
- no associated donor site morbidity;
- possibility of obtaining sufficient amount of tissue from the site adjacent to the defect;
- adequate vascularity of the flap with minimal risk of infection, necrosis, and graft rejection;
- minimal postoperative complications;
- better patient satisfaction.

Thus, this technique offers a successful and viable alternative for the coverage of localized gingival recessions with an inadequate vestibular depth. However, the limitation of the technique remains as it can only be used for single-tooth recession coverage and requires great surgical proficiency.
